# Supplementary material for: Small, solubilized platinum nanocrystals consist of an ordered core surrounded by mobile surface atoms
Source: Commun Chem. 2024 Jan 3;7:4. doi: 10.1038/s42004-023-01087-x (PMC10764312; doi:10.1038/s42004-023-01087-x)
Supplement: Supplementary file 2 — Supplementary Information [file 42004_2023_1087_MOESM2_ESM.pdf]

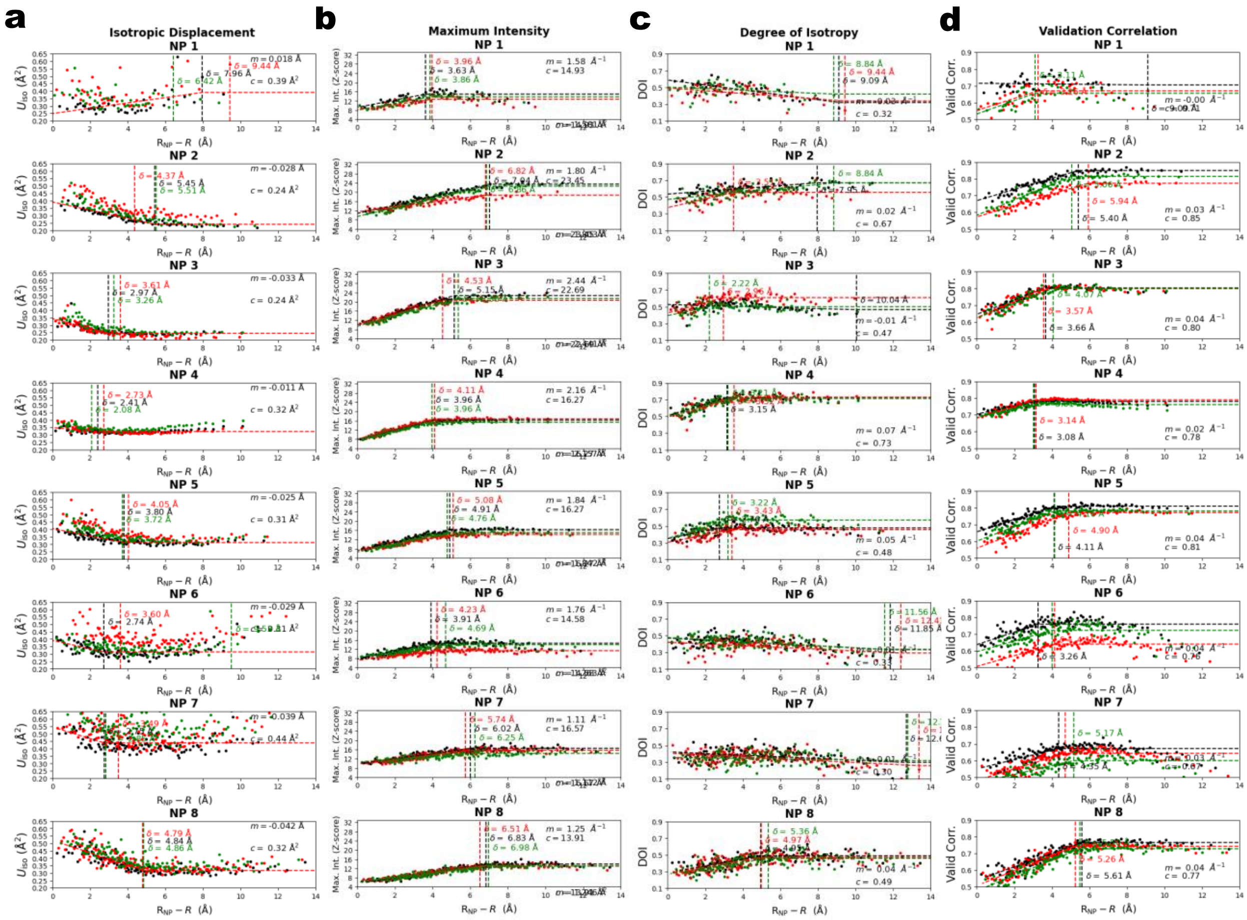


**Supplementary Figure 1.** Penetration depth plots for various atomic statistics. (a) Isotropic displacement, (b) maximum intensity, (c) DOI (Degree Of Isotropy), and (d) validation correlation versus radial depth for each of the eight NPs. Each point is an average of five atoms with similar radial positions. Red dashed lines show fits using Equation 1. Vertical dashed lines indicate the penetration depth $\delta$ estimated from the fit. Red, green, and black data points correspond to *part1*, *part2*, and *merged*, respectively.
